# Long‐term effects of adolescent sport experience, DRD2 and COMT genes, and their interaction on sport participation in adulthood

**DOI:** 10.1002/brb3.2459

**Published:** 2021-12-14

**Authors:** Chung Gun Lee, Hyoyoul Moon, Joon‐Ho Kang, Joo Hee Choi, Ju Hyuk Kwon

**Affiliations:** ^1^ Department of Physical Education, College of Education Seoul National University 1 Gwanak‐ro, Gwanak‐gu Seoul South Korea; ^2^ Institute of Sport Science Seoul National University 1 Gwanak‐ro, Gwanak‐gu Seoul South Korea

**Keywords:** adolescent sport experience, adulthood, COMT, DRD2, gene‐by environment interactions, sport participation

## Abstract

**Background:**

The present study investigated the joint impact of adolescent sport experience and dopamine‐related genes (i.e., DRD2 and COMT genes) on sport participation in adulthood.

**Methods:**

Using the National Longitudinal Study of Adolescent Health (Add Health) data, the hierarchical multivariable logistic regression models for predicting sport participation in wave 3 (around 20 years of age) and wave 4 (around 30 years of age) were conducted separately by gender (male and female) and gene (DRD2 and COMT genes).

**Results:**

Adolescent sport experience significantly interacted with the number of DRD2 A1 alleles and COMT Met alleles in affecting wave 3 sport participation among male adults. The interaction between adolescent sport experience and DRD2 gene significantly affected wave 4 sport participation in opposite direction to that affected wave 3 sport participation among male participants. Among female participants, there were no significant interaction effects between dopamine‐related genes and adolescent sport experience on sport participation in both wave 3 and 4.

**Conclusions:**

Since adult sport participation is most likely to be influenced by the joint impact of environmental and genetic factors, it is important to consider gene‐by‐environment interactions when designing policies or programs to promote adult sport participation.

## Graphical abstract

The significant effects of the number of DRD2 A1 alleles on wave 3 sport participation became insignificant if male participants actively participated in sports in adolescence, indicating that adolescent sport experience may overpower the effect of DRD2 genes on sport participation in adults aged around 20 years.

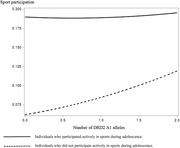

## 1 INTRODUCTION

One of the most effective ways to increase regular physical activity is to participate in sporting activities. Sports participation invariably involves physical activity and inherently includes various enjoyable aspects, such as personal challenge, social interaction, goal achievement, and competition. The International Society for Physical Activity and Health also suggests that sports participation is “an investment that works” to increase physical activity and improve health (Trost et al., 2014). Performing physical activity has been shown to be associated with a lower risk of developing various chronic diseases, such as hypertension, various types of cancer, coronary heart disease, stroke, osteoporosis, and Type 2 diabetes mellitus (U.S. Department of Health & Human Services, 2020). Engaging in physical activity also promotes psychological well‐being by reducing psychological distress and enhancing self‐esteem (U.S. Department of Health & Human Services, 2020). Despite these well‐known benefits of physical activity, the sport participation rate has been shown to peak in late adolescence and decrease throughout adulthood in many countries including the United States (Australian Bureau of Statistics, 2012; Birchwood et al., 2008; Eime et al., 2016; Lee et al., 2020; Maia et al., 2010). Therefore, the development of efficient programs or policies that can help increase and maintain sport participation rate throughout adulthood is becoming critically important. However, numerous studies have been carried out on the modification and maintenance of physical activity‐related behaviors in the adult population in the past decades and the results of these studies were unsatisfactory (Marcus et al., 2006; Müller‐Riemenschneider et al., 2008). These results imply that there needs to be a new approach to understand sport participation behavior in adulthood.

Individual genomic variations have recently got momentous attention in addressing physical activity‐related behaviors among adults. There is a growing body of studies emphasizing the role of dopamine‐related genes in physical activity‐related behaviors because the neurotransmitter dopamine affects the responsivity of the brain reward system and the way humans learn (Wise, 2004). Simonen et al. examined the relationship between dopamine receptor D2 (DRD2) gene and physical activity among black and white adults. They found out that DRD2 TT homozygotes were significantly less likely to participate in sports and physical activity than DRD2 CT heterozygotes and CC homozygotes in White women (Simonen et al., 2003). Similarly, Flack et al. showed that carrying the A1 allele of DRD2 gene is associated with a lower RRVexercise (relative reinforcing value of exercise) among 178 adults (127 were female) (Flack et al., 2019). Lee et al. also found out that the more women possess the DRD2 A1 allele, the more likely they are not to participate in sports throughout adolescence and young adulthood (Lee et al., 2020). Since A1 allele and T allele of DRD2 gene have been shown to be associated with several additive behaviors (Blum et al., 2017; Deng et al., 2015; Meyers et al., 2013; Ohmoto et al., 2013; Wang et al., 2016), women who carry these alleles may engage in addictive behaviors rather than sports in order to satisfy themselves. Among men, on the contrary, the more they carry the DRD2 A1 allele, the more likely they are to participate in sports throughout adolescence and young adulthood (Lee et al., 2020), indicating that exercise and sport participation can become addictive in the male population (Villella et al., 2011). The catechol‐O‐methyltransferase (COMT) gene is also one of the strongest candidate genes for physical activity‐related behaviors and has been shown to be associated with several addictive behaviors (Kauhanen et al., 2000; Munafò et al., 2008; Schellekens et al., 2013). Rosso et al. examined whether intervention‐induced changes in physical activity differ according to dopamine‐related genes including the COMT gene. The differences in physical activity between intervention and control groups were greater for COMT methionine (Met) homozygotes than for those with at least one COMT valine (Val) allele (Rosso et al., 2018). Van der Mee et al. found out that externally paced exercise behavior was positively associated with COMT Met allele among adult population. They also found out that this association was even stronger among participants who regularly participate in exercise and sporting activities (Van der Mee et al., 2018). The results of these studies strongly suggest that current/previous exercise and sport experiences may act as a trigger for increasing or maintaining voluntary exercise and sport participation in later life among individuals with DRD2 A1 allele and/or COMT Met allele. On the other hand, there are studies showing that there is no significant association of DRD2 and COMT genes with physical activity‐related behaviors in adults (De Moor et al., 2009; Flack et al., 2019; Huppertz et al., 2014; Jozkow et al., 2013). However, these studies did not consider current/previous exercise and sport experiences, did not analyze separately by gender, and were cross‐sectional studies that tested the effects of genes on physical activity at some point in time.

Since human behaviors are most likely to be influenced by the joint impact of environmental and genetic factors (Halldorsdottir & Binder, 2017; Young‐Wolff et al., 2011), gene‐by‐environment interactions should be considered when predicting sport participation. In previous literature, there is some evidence showing that participating in physical activity in adolescence increases the probability of participation in physical activity in adulthood (Batista et al., 2019). Moreover, the association between adolescence and adulthood physical activity is greater if the type of physical activity is related to sports (Bélanger et al., 2015; Kjønniksen et al., 2008). Using the National Longitudinal Study of Adolescent Health (Add Health) data, the present study investigated the joint impact of adolescent sport experience and dopamine‐related genes (i.e., DRD2 and COMT genes) on sport participation in adulthood because the results of previous genetic studies imply that there are potential interactions between current/previous exercise and sport experiences and dopamine‐related genes in the prediction of voluntary exercise and sport participation.

## 2 METHODS

### 2.1 Data

The present study used the Add Health data. It is a prospective longitudinal study that followed up a nationally representative sample of middle and high school students in the United States. All high schools in the United States with more than 30 enrolled students were included in the primary sampling frame for Add Health. Using systematic random sampling, a total of 80 high schools were selected after stratification by school type, size, ethnic mix, region, and urbanicity. Approximately 70% of these 80 high schools were recruited. Middle schools in the United States that sent graduates to recruited high schools were also recruited. The final sample consisted of 134 middle and high schools in the United States. Students were randomly selected from each recruited school after stratification by age and grade. The wave 1 survey was conducted in 1995 and a total of 20,745 adolescents were surveyed in their homes (79% response rate). The wave 2 follow‐up survey was conducted 1 year after wave 1 (89% response rate). The wave 3 (77% response rate) and wave 4 (80% response rate) follow‐up surveys were conducted 6 and 13 years after wave 1, respectively. More information about the survey design of Add Health can be found elsewhere (Harris et al., 2019). The participants for the present study were those who provided their genetic information (i.e., COMT and DRD2 genes) at wave 4. The data that support the findings of this study are available on request from the corresponding author. The data are not publicly available due to privacy or ethical restrictions.

### 2.2 Measures

Adolescent sport experience was measured by asking respondents to indicate the number of times per week they played an active sport, such as softball, basketball, baseball, football, swimming, or soccer in waves 1 and 2. Respondents who responded “more than 4 days per week” in wave 1 or 2 were defined as a person who actively engaged in sports during adolescence. Sport participation in young adulthood was assessed by asking respondents to indicate the number of times per week they participated in individual (e.g., cycle racing, running, wrestling, cross‐country skiing, swimming, or martial arts) or strenuous team sports (e.g., field hockey, football, soccer, lacrosse, basketball, rugby, or ice hockey) in waves 3 and 4. Respondents who participated in individual or team sports more than 4 days per week in each wave were categorized as active participators.

Using a salivary DNA collection device (OrageneTM DNA genotek, Ottawa, Ontario, Canada), participants provided 2 ml of saliva in wave 4. In order to extract DNA, saliva was packed in the container (SafTpak #STP‐210, Edmonton, Alberta, Canada) and shipped to the University of Colorado, Institute for Behavioural Genetics. Nine (0.06%) were empty and 24 (0.15%) were damaged or leaking during shipping. Further information about Add Health wave 4 candidate gene data is reported in more detail elsewhere (Smolen et al., 2013).

Single nucleotide polymorphisms (SNPs) were genotyped on either an Applied Biosystems TaqMan® OpenArray® (archived samples) or an Illumina BeadXpress® GoldenGate® (non‐archived samples) platform for both DRD2 Taq1A SNP rs1800497 in the 30 UTR and COMT val158met SNP rs4680. The DRD2 Taq1A (rs1800497) assay and COMT val158met (rs4680) assay were carried out using a fluorogenic 5′nuclease (Taqman®, ABI, Foster City, CA) method (Haberstick & Smolen, 2004). These were done on an ABI Prism® 7000 Sequence Detection System using the allelic discrimination mode (Livak, 1999). Since the A1 allele of the Taq1A polymorphism (rs1800497) and Met allele of the val158met polymorphism (rs4680) have been associated with exercise and sports participation in adults, we used the number of DRD2 A1 alleles (0 to 2) and COMT Met alleles (0 to 2) to predict sport participation in young adulthood.

Additional covariates were age, gender (male and female), education (high school or less, college, and graduate school or more). Since addictive behaviors have been shown to be associated with DRD2 and COMT genes, smoking (current smoker), binge drinking (5 or more drinks in a row), and marijuana use (1 or more times) were also used as a covariate when predicting sport participation in adulthood.

### 2.3 Statistical analysis

The hierarchical multivariable logistic regression models for predicting sport participation in wave 3 (around 20 years of age) and wave 4 (around 30 years of age) were conducted separately by gender (male and female) and gene (DRD2 and COMT genes). In model 1, the effect of gene on adult sport participation was examined after controlling for age, education, and addictive behaviors. In model 2, adolescent sport experience was included in model 1 to see if there is any change in the effect of gene on sport participation. In model 3, the interaction between adolescent sport experience and gene was included in model 2 to examine the joint impact of adolescent sport experience and gene on adult sport participation. Wave 3 Sport participation was included as a covariate in all models predicting wave 4 sport participation. Other covariates (i.e., age, education, and addictive behaviors) were assessed at the same wave as dependent variables. Deviance statistics (‐2 log likelihood) were used to compare nested models. The *p*‐values lower than .05 were considered as significant. Analysis of variance (ANOVA) and Chi‐square test were used to compare between active and inactive participators in sports. All the analyses were performed using SAS version 9.4 (SAS Institute Inc., Cary, NC).

## 3 RESULTS

### 3.1 Descriptive statistics

Table 1 presents characteristics of female and male participants by sport participation in wave 3. The mean age of male participants at wave 3 was 22.03 years. Male participants with higher levels of education were more likely to be an active participator in sports. Current smokers and marijuana users were less likely to be an active participator in sports. Adolescent sport experience was positively associated with wave 3 sport participation in male participants. The number of DRD2 A1 alleles was also positively associated with wave 3 sport participation. The mean age of female participants at wave 3 was 21.83 years. Unlike male participants, binge drinkers were more likely to be active participators in sports among female participants. Marijuana use and the number of DRD2 A1 alleles were not associated with sport participation among female participants in wave 3.

**TABLE 1:** Characteristics of participants by sport participation in wave 3 (*n*= 15,699)

		Sport participation in wave 3		
Characteristics	Total sample	Active participator	Inactive participator	*p*‐Value
*Male* (*n* = 7,352)				
Mean age (*SD*)	22.03 (1.76)	21.62 (1.79)	22.09 (1.75)	<.0001
Education (%)				.0032
High school or less	2900 (48.87)	330 (43.19)	2570 (49.71)	
College	2939 (49.53)	419 (54.84)	2520 (48.74)	
Graduate school or more	95 (1.60)	15 (1.96)	80 (1.55)	
Smoking (%)				<.0001
Current smoker	2045 (34.57)	192 (25.26)	1853 (35.94)	
Non‐smoker	3871 (65.43)	568 (74.74)	3303 (64.06)	
Binge drinking (%)				.1917
Binge drinker	3360 (56.80)	415 (54.61)	2945 (57.12)	
Non‐binge drinker	2556 (43.20)	345 (45.39)	2211 (42.88)	
Marijuana use (%)				.0001
Marijuana user	1594 (26.98)	161 (21.24)	1433 (27.82)	
Non‐marijuana user	4315 (73.02)	597 (78.76)	3718 (72.18)	
Adolescent sport participation (%)				<.0001
Active participator	2643 (53.23)	499 (73.71)	2144 (50.00)	
Inactive participator	2322 (46.77)	178 (26.29)	2144 (50.00)	
DRD2 (%)				.0467
A1 (‐) group	2985 (53.35)	371 (51.39)	2614 (53.64)	
A1 (+) group	2199 (39.30)	282 (39.06)	1917 (39.34)	
A1 (++) group	411 (7.35)	69 (9.56)	342 (7.02)	
COMT (%)				.7382
Met (‐) group	1745 (32.43)	230 (33.00)	1515 (32.35)	
Met (+) group	2565 (47.68)	336 (48.21)	2229 (47.60)	
Met (++) group	1070 (19.89)	131 (18.79)	939 (20.05)	
Total (%)	5938	764 (12.87)	5174 (87.13)	
*Female* (*n* = 8,347)				
Mean age (*SD*)	21.83 (1.75)	333 (21.44)	6732 (21.84)	<.0001
Education (%)				<.0001
High school or less	3009 (42.60)	100 (30.12)	2909 (43.21)	
College	3911 (55.37)	218 (65.66)	3693 (54.86)	
Graduate school or more	144 (2.04)	14 (4.22)	130 (1.93)	
Smoking (%)				.0003
Current smoker	2037 (28.90)	67 (20.12)	1970 (29.33)	
Non‐smoker	5012 (71.10)	266 (79.88)	4746 (70.67)	
Binge drinking (%)				.0011
Binge drinker	2781 (39.49)	160 (48.05)	2,621 (39.07)	
Non‐binge drinker	4261 (60.51)	173 (51.95)	4088 (60.93)	
Marijuana use (%)				.8409
Marijuana user	1214 (17.22)	56 (16.82)	1158 (17.24)	
Non‐marijuana user	5835 (82.78)	277 (83.18)	5558 (82.76)	
Adolescent sport participation (%)				<.0001
Active participator	1605 (28.18)	158 (52.84)	1447 (26.82)	
Inactive participator	4090 (71.82)	141 (47.16)	3949 (73.18)	
DRD2 (%)				.3989
A1 (‐) group	3658 (54.77)	183 (57.55)	3475 (54.63)	
A1 (+) group	2508 (37.55)	116 (36.48)	2392 (37.60)	
A1 (++) group	513 (7.68)	19 (5.97)	494 (7.77)	
COMT (%)				.6405
Met (‐) group	2014 (31.28)	98 (31.31)	1916 (31.28)	
Met (+) group	3040 (47.22)	154 (49.20)	2886 (47.12)	
Met (++) group	1384 (21.50)	61 (19.49)	1323 (21.60)	
Total (%)	7067	333 (4.71)	6734 (95.29)	

Abbreviations: DRD2 , dopamine receptor D2; COMT , catechol‐O‐methyltransferase; *SD*, standard deviation.

Missing data were excluded in calculating the percentage.

The characteristics of male and female participants by sport participation in wave 4 are presented in Table 2. The mean age of male and female participants at wave 4 were 28.59 and 28.38 years, respectively. Individuals with higher levels of education were more likely to participate in sports among both male and female participants. Current smokers were less likely to participate in sports among both genders. Binge drinkers were more likely to be an active participator in sports among female participants only. Adolescent sport experience was positively associated with wave 4 sport participation in both genders. Both DRD2 and COMT genes were not associated with wave 4 sport participation among both genders.

**TABLE 2:** Characteristics of participants by sport participation in wave 4 (*n* =15,699)

		Sport participation in wave 4		
Characteristics	Total sample	Active participator	Inactive participator	*p*‐Value
*Male* (*n* = 7352)				
Mean age (*SD*)	28.59 (1.77)	28.49 (1.75)	28.60 (1.78)	.1126
Education (%)				<.0001
High school or less	2870 (39.10)	210 (29.58)	2660 (40.11)	
College	3791 (51.64)	408 (57.46)	3383 (51.02)	
Graduate school or more	680 (9.26)	92 (12.96)	588 (8.87)	
Smoking (%)				<.0001
Current smoker	2595 (35.46)	195 (27.50)	2400 (36.31)	
Non‐smoker	4724 (64.54)	514 (72.50)	4210 (63.69)	
Binge drinking (%)				.6890
Binge drinker	3989 (54.61)	380 (53.90)	3609 (54.69)	
Non‐binge drinker	3315 (45.39)	325 (46.10)	2990 (45.31)	
Marijuana use (%)				.3500
Marijuana user	1494 (20.38)	135 (19.04)	1359 (20.53)	
Non‐marijuana user	5835 (79.62)	574 (80.96)	5261 (79.47)	
Adolescent sport participation (%)				<.0001
Active participator	3239 (53.84)	420 (67.96)	2819 (52.22)	
Inactive participator	2777 (46.16)	198 (32.04)	2579 (47.78)	
DRD2 (%)				.3659
A1 (‐) group	3740 (54.50)	345 (52.51)	3395 (54.71)	
A1 (+) group	2627 (38.28)	257 (39.12)	2370 (38.20)	
A1 (++) group	495 (7.21)	55 (8.37)	440 (7.09)	
COMT (%)				.1282
Met (‐) group	2125 (32.19)	222 (35.81)	1903 (31.82)	
Met (+) group	3171 (48.04)	283 (45.65)	2888 (48.29)	
Met (++) group	1305 (19.77)	115 (18.55)	1190 (19.90)	
Total (%)	7343	710 (9.67)	6633 (90.33)	
*Female* (*n* = 8,347)				
Mean age (*SD*)	28.38 (1.77)	28.21 (1.82)	28.39 (1.76)	.0619
Education (%)				<.0001
High school or less	2490 (29.86)	65 (18.62)	2425 (30.35)	
College	4624 (55.45)	204 (58.45)	4420 (55.32)	
Graduate school or more	1225 (14.69)	80 (22.92)	1145 (14.33)	
Smoking (%)				.0265
Current smoker	2342 (28.15)	80 (22.92)	2262 (28.38)	
Non‐smoker	5977 (71.85)	269 (77.08)	5708 (71.62)	
Binge drinking (%)				.0035
Binge drinker	3297 (39.64)	164 (47.13)	3133 (39.31)	
Non‐binge drinker	5021 (60.36)	184 (52.87)	4837 (60.69)	
Marijuana use (%)				.6440
Marijuana user	1009 (12.10)	45 (12.89)	964 (12.07)	
Non‐marijuana user	7327 (87.90)	304 (87.11)	7023 (87.93)	
Adolescent sport participation (%)				<.0001
Active participator	1839 (27.99)	135 (47.04)	1704 (27.12)	
Inactive participator	4731 (72.01)	152 (52.96)	4579 (72.88)	
DRD2 (%)				.2327
A1 (‐) group	4287 (54.90)	196 (59.39)	4091 (54.70)	
A1 (+) group	2929 (37.51)	110 (33.33)	2819 (37.69)	
A1 (++) group	593 (7.59)	24 (7.27)	569 (7.61)	
COMT (%)				.1895
Met (‐) group	2340 (31.10)	87 (26.85)	2253 (31.30)	
Met (+) group	3595 (47.79)	169 (52.16)	3426 (47.59)	
Met (++) group	1588 (21.11)	68 (20.99)	1520 (21.11)	
Total (%)	8339	349 (4.19)	7990 (95.81)	

Abbreviations: DRD2, dopamine receptor D2; COMT, catechol‐O‐methyltransferase; *SD*, standard deviation.

Missing data were excluded in calculating the percentage.

### 3.2 Multivariable logistic regression models

**FIGURE 1:**
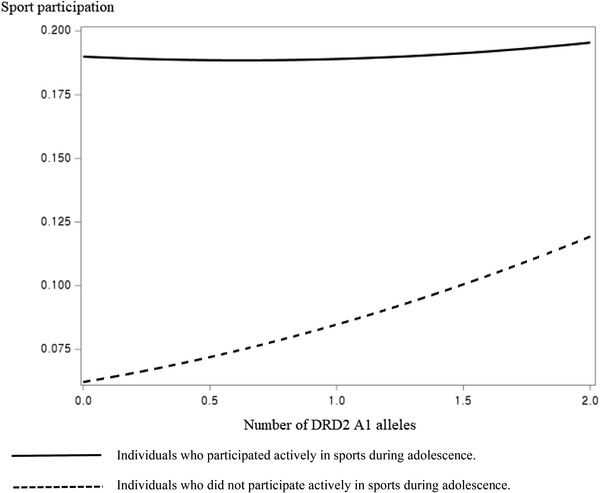
The effect of interaction between DRD2 and adolescent sport experience on wave 3 sport participation

**FIGURE 2:**
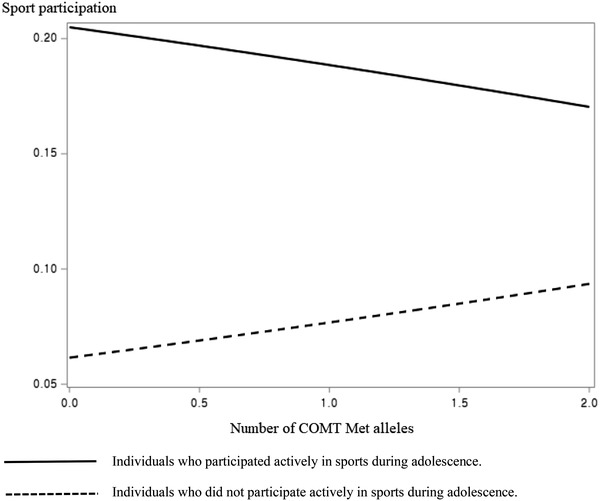
The effect of interaction between COMT and adolescent sport experience on wave 3 sport participation

The results of multivariable logistic regression for predicting sport participation in wave 3 (around 20 years of age) are presented in Table 3. The number of DRD2 A1 alleles (marginally) significantly affected wave 3 sport participation in model 1 among male participants. This effect became insignificant after adjustment for adolescent sports experience (model 2), which means that the effect of the DRD2 gene on wave 3 sport participation may be moderated by adolescent sport experience. In model 3, there was a significant interaction effect between DRD2 gene and adolescent sport experience on wave 3 sport participation. Male participants' sport participation in wave 3 were not affected by the DRD2 gene if they participated actively in sports during adolescence, whereas the number of DRD2 A1 alleles had a significant positive effect on wave 3 sport participation among male participants who did not participate actively in sports during adolescence (see Figure 1). The effect of COMT gene on wave 3 sport participation in male participants was similar to that of the DRD2 gene. Although the number of COMT Met alleles had a positive effect on sport participation in wave 3 if participants did not participate actively in sports during adolescence, this effect disappeared if participants were an active participator in sports in adolescence (see Figure 2). There was no significant interaction effect between genes and adolescent sport experience on wave 3 sport participation among female participants.

**TABLE 3:** Multivariable logistic regression for predicting wave 3 sport participation

	DRD2 A1 allele						COMT Met allele					
	Model 1		Model 2		Model 3		Model 1		Model 2		Model 3	
Independent variables	Coef.	(*SE*)	Coef.	(*SE*)	Coef.	(*SE*)	Coef.	(*SE*)	Coef.	(*SE*)	Coef.	(*SE*)
*Male*												
Gene (number of alleles)	0.115	(0.063)*	0.103	(0.069)	0.337	(0.125)***	−0.037	(0.058)	0.003	(0.063)	0.226	(0.117)*
Adolescent sport experience			1.008	(0.097)***	1.206	(0.135)***			1.009	(0.099)***	1.295	(0.164)***
Gene × Adolescent sport experience					−0.333	(0.150)**					−0.314	(0.138)**
−2 log likelihood	4158.142		3496.531		3491.624		4010.087		3368.032		3362.879	
*Female*												
Gene (Number of alleles)	−0.099	(0.094)	−0.046	(0.099)	−0.010	(0.138)	−0.057	(0.082)	−0.054	(0.088)	−0.029	(0.123)
Adolescent sport experience			0.968	(0.127)***	1.005	(0.161)***			0.997	(0.128)***	1.042	(0.202)***
Gene × Adolescent sport experience					−0.074	(0.198)					−0.050	(0.175)
−2 log likelihood	2477.220		2100.856		2100.716		2424.999		2047.745		2047.664	

All models were adjusted for age, education, and addictive behaviors.

Abbreviations: Coef., logit coefficient; *SD*, standard deviation.

* *p* < .1, **p < .05, ***p < .01.

**FIGURE 3:**
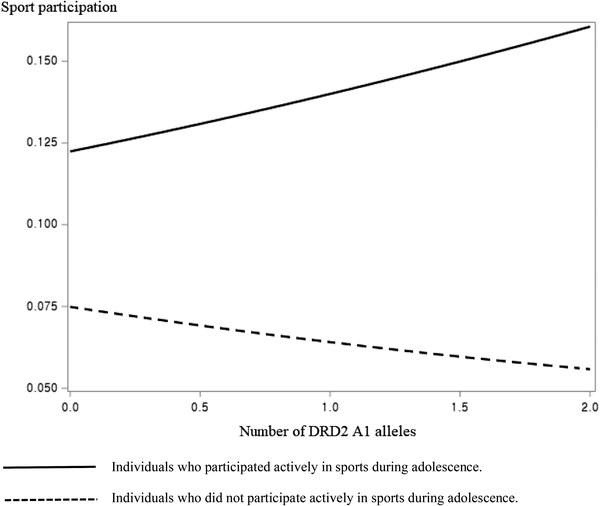
The effect of interaction between DRD2 and adolescent sport experience on wave 4 sport participation

Table 4 presents the results of logistic regression for predicting sport participation in wave 4 (around 30 years of age). The number of DRD2 A1 alleles did not affect wave 4 sport participation in model 1 among male participants. Adolescent sport experience still had a significant effect on wave 4 sport participation in model 2, indicating that sport participation in adolescence has a long‐term effect on sport participation in adulthood (around 30 years of age). Sport participation at wave 3 also had a significant effect on wave 4 sport participation in both gender groups. There was a significant interaction effect between the number of DRD2 A1 alleles and adolescent sport experience on wave 4 sport participation in model 3. Sport participation in wave 4 was significantly affected by the number of DRD2 A1 alleles if male participants participated actively in sports in adolescence, whereas DRD2 gene did not have any significant effect on wave 4 sport participation if male participants were not active participators in sports during adolescence (see Figure 3), indicating that adolescent sport experience may act as a trigger for increasing sport participation among adults aged around 30 years with DRD2 A1 allele. There was no significant interaction effect between the COMT gene and adolescent sport experience on wave 4 sport participation among male participants. Similar to the results of logistic regression models for predicting wave 3 sport participation, the interaction effects between genes and adolescent sport experience on wave 4 sport participation were not significant among female participants.

**TABLE 4:** Multivariable logistic regression for predicting wave 4 sport participation

	DRD2 A1 allele						COMT Met allele					
	Model 1		Model 2		Model 3		Model 1		Model 2		Model 3	
Independent variables	Coef.	(*SE*)	Coef.	(*SE*)	Coef.	(*SE*)	Coef.	(*SE*)	Coef.	(*SE*)	Coef.	(*SE*)
*Male*												
Gene (number of alleles)	0.011	(0.074)	0.048	(0.079)	−0.196	(0.142)	−0.096	(0.068)	−0.096	(0.072)	−0.067	(0.122)
Adolescent sport experience			0.512	(0.107)***	0.317	(0.139)**			0.491	(0.110)***	0.528	(0.170)***
Wave 3 sport participation			1.157	(0.113)***	1.165	(0.113)***			1.152	(0.116)***	1.151	(0.116)***
Gene × Adolescent sport experience					0.360	(0.171)**					−0.043	(0.151)
−2 log likelihood	3322.767		2899.609		2895.074		3158.925		2752.530		2752.449	
*Female*												
Gene (Number of alleles)	−0.057	(0.100)	−0.064	(0.110)	−0.071	(0.149)	0.087	(0.088)	0.089	(0.096)	0.154	(0.130)
Adolescent sport experience			0.666	(0.142)***	0.658	(0.178)***			0.691	(0.143)***	0.826	(0.231)***
Wave 3 sport participation			1.292	(0.186)***	1.292	(0.186)***			1.289	(0.187)***	1.289	(0.187)***
Gene × Adolescent sport experience					0.016	(0.222)					−0.143	(0.192)
−2 log likelihood	2190.043		1800.554		1800.549		2141.246		1765.608		1765.053	

All models were adjusted for age, education, addictive behaviors, and wave 3 sport participation.

Abbreviations: Coef., logit coefficient; *SD*, standard deviation.

** *p* < .05, ***p < .01.

## 4 DISCUSSION

To our knowledge, the present study is the first study that investigated the joint impact of adolescent sports experience and dopamine‐related genes (i.e., DRD2 and COMT genes) on sport participation in adulthood. The results of the present study showed that adolescent sport experience is strongly associated with sport participation not only among adults aged around 20 years but also among adults around 30 years of age. This result is in line with previous findings showing that participating in sports during adolescence increases the probability of sports participation in adulthood (Batista et al., 2019; Bélanger et al., 2015; Kjønniksen et al., 2008), emphasizing the great importance of adolescent sport experience because of its strong long‐term effect on sport participation in adulthood. The strong effects of sport participation at wave 3 on wave 4 sport participation in both gender groups are also in line with a previous study showing that individuals with high levels of exercise in young adulthood become higher exercisers in adulthood (Van der Zee et al., 2019). More importantly, this study also found out that there are significant interaction effects between adolescent sports experience and dopamine‐related genes on sport participation in adulthood.

The present study found out that adolescent sport experience interacts with the number of DRD2 A1 alleles and COMT Met alleles in affecting sport participation among male adults around 20 years of age. As shown in Figures 1 and 2, the number of both DRD2 A1 alleles and COMT Met alleles significantly affected wave 3 sport participation among male participants who did not actively participate in sports in adolescence. The A1 allele of DRD2 gene is shown to be associated with lower DRD2 density, lower inhibitory feedback, and, therefore, higher synaptic dopamine levels (Huppertz et al., 2014; Rosso et al., 2018). The Met allele of COMT gene is associated with slower dopamine clearance and, consequently, is related to higher synaptic dopamine levels (Huppertz et al., 2014; Rosso et al., 2018). There may have been a lot of opportunities for male participants in wave 3 to participate in a diverse range of sports because most of them were still high school or college students (see Table 1), and male participants with higher synaptic dopamine levels may have participated more in sports because of their higher reward sensitivity. On the contrary, significant effects of the number of DRD2 A1 alleles and COMT Met alleles on wave 3 sport participation became insignificant if male participants actively participated in sports in adolescence (see Figures 1 and 2), indicating that adolescent sport experience may overpower the effect of DRD2 and COMT genes on sport participation in adults aged around 20 years. This result imply that sport participation during adolescence not only has a long‐term effect but also has a stronger effect than genes on sport participation in adults around 20 years of age. Since the importance of adolescent sport participation can never be emphasized enough (Telama et al., 2005), middle and high school physical education programs need to be mainly designed to promote sport participation among adolescents.

Another interesting finding in this study was that the interaction between adolescent sport experience and DRD2 gene affected wave 4 sport participation in the opposite direction to that affected wave 3 sport participation among male participants. The number of DRD2 A1 alleles did not affect wave 4 sport participation among male participants who were not active participators in sports in adolescence (see Figure 3). This result is consistent with a previous study showing that the magnitude of genetic influence on exercise‐related behaviors decreases with age (Vink et al., 2011). Since male participants in wave 4 were aged around 30 years, they may have had less leisure time to participate in sports than high school or college students. They may have been busy with job‐related duties, which means that the effect of DRD2 gene on sport participation may be overpowered by environmental constraints among male participants aged around 30 years. However, the effect of the number of DRD2 A1 alleles on wave 4 sport participation was found to be significant among male participants who were active participators in sports during adolescence (see Figure 3). This result can be interpreted as a triggering effect of adolescent sport experiences on the DRD2 gene because previous studies have shown that current or previous exercise experiences may act as triggers for increasing voluntary exercise participation in later life among people who carry the DRD2 A1 allele and/or COMT Met allele (Lee et al., 2020; Rosso et al., 2018; Van der Mee et al., 2018).

Among female participants, there were no significant interaction effects between dopamine‐related genes and adolescent sport experience on sport participation in both waves 3 and 4. Previous studies have shown that women are more susceptible to socio‐cultural norms and perceptions compared to men because their social relationships are characterized by emotional support, self‐disclosure, and intimacy (Maccoby, 1990; Rose & Rudolph, 2006). Their higher susceptibility to environment may, therefore, disguise the effect of dopamine‐related genes on sport participation. This result suggests that dopamine‐related genes are more appropriate candidate genes for adult sport participation in male participants rather than female participants.

The present study has several limitations. First, more detailed information on sport participation is needed because there exist many different types of sporting activity. In order to understand the influence of dopamine‐related genes on sport participation in more detail, future studies should at least distinguish team sport participation from individual sport participation. Second, since this study used self‐reported sport participation, there is a risk of recall bias. Third, since Add Health is a prospective longitudinal study that followed up a nationally representative sample of middle and high school students in the United States, the results of this study may not be generalizable to other countries. Despite these limitations, the present study may contribute to the literature by providing new information on the joint impact of adolescent sport experience and dopamine‐related genes on adult sport participation. Our results suggest that adolescent sport experience may overpower the effect of DRD2 and COMT genes on sport participation in male adults aged around 20 years. However, our results also indicate that adolescent sport experience may act as a trigger for increasing sport participation in male adults around 30 years of age, who carry the DRD2 A1 allele. Since adult sport participation is most likely to be influenced by the joint impact of environmental and genetic factors, it is important to consider gene‐by‐environment interactions when designing policies or programs to promote adult sport participation.

### PEER REVIEW

The peer review history for this article is available at https://publons.com/publon/10.1002/brb3.2459

## Data Availability

The data on which this study is performed are available on request from the corresponding author. The data are not publicly available due to privacy or ethical restrictions.
